# Reply to ‘Comment on “Validation of a contemporary prostate cancer grading system using prostate cancer death as outcome”’

**DOI:** 10.1038/bjc.2016.347

**Published:** 2016-10-25

**Authors:** Daniel M Berney

**Affiliations:** 1 Department of Molecular Oncology, Barts Cancer Institute Queen Mary University of London, London, UK

**Sir,**

I thank Dell’oglio *et al* for their comments and interest in our paper (Berney *et al*, 2016), and their generally supportive comments. The authors conclude that the new grading system is merely a ‘user-friendly instrument’, however, it includes a number of pattern refinements based on previous work outlined fully in our article (Berney *et al*, 2016), including the fact that all cribriform glands and glomeruloid glands should be assigned a Gleason pattern 4 regardless of morphology, and the grading of mucinous carcinoma of the prostate should be based on its underlying growth pattern rather than grading them all as pattern (Epstein *et al*, 2016).

They ask whether the new prostate cancer grading system improves the prediction of prostate cancer death relative to the standard grading system. The problem with this question is that there is divergence on interpretation of the standard model. The revisions to the Gleason system have been driven by a lack of clarity in pattern assignment, particularly, but not exclusively centred on grade assignation in cribriform lesions, which have been variably assigned and interpreted (Dong *et al*, 2013; Kir *et al*, 2014; Kweldam *et al*, 2014). It is virtually impossible to compare the new grading system with variable interpretations of ISUP 2005, and more recent suggested revisions of pattern present in the 2005 classification (Epstein, 2010). Recently, this variable interpretation was examined in detail by the European Network of Uro-Pathology (Berney *et al*, 2013). The paper cited by the authors, which found no improvement in grading by grade groups, included no reassignment of grades based on these clarifications, but was a retrospective exercise mining old reports (Loeb *et al*, 2016). Such retrospective mining of old reports should always be treated with caution.

The second to fourth criticisms of Dell’Oglio *et al* are related to the cohort itself. Unfortunately the ‘perfect’ prostate cohort does not exist. No cohort can have both long follow-up and be to contemporary standards of diagnosis and treatment. The radical prostatectomy data is self-evidently not available at diagnosis.

Two criticisms by Dell’Oglio *et al* of our cohort are virtually mutually exclusive suggestions: not using radical prostatectomy as a more accurate assessment of Gleason score, and not including patients over 76.

The most vital questions we have to answer for the treatment of prostate cancer remains in the clinic prior to any proposed treatment. Conservative management is a reasonable surrogate for the natural history of prostate cancer and an indication of how many men are being treated unnecessarily. At present few other comparable cohorts have been published with long-term outcome available.

Finally, we consider the importance of a patient-centered system is not a trivial one, especially one that does improve the groupings considered for future analysis. Grade Group 1 out of 5 better conveys indolent behavior compared with a GS of 6 out of 10, and will permit more rational and less emotional decision-making in regards to active surveillance. Grade Group 2 out of 5 (as opposed to Gleason score 7 out of 10) has a very good prognosis with rare metastases. Grade Group 3 has a significantly worse prognosis than Grade 2 as opposed to Gleason score 7, which combines Gleason scores 3+4 and 4+3 and is often incorrectly combined together for treatment and prognosis protocols. Grade Group 4 out of 5 is not considered the highest grade (as opposed to the often combined together Gleason scores 8–10) and has a significantly better prognosis than Grade Group 5 (Gleason scores 9–10). Finally, Grade Group 5 obviates the need to distinguish between Gleason scores 4+5, 5+4 and 5+5 just as Grade Group 1 makes irrelevant, the distinction between Gleason scores 2+2, 2+3, 3+2 and 3+3. These practical points are likely to be of immense use in patient management and their comprehension of the severity of their disease.

## References

[bib1] Berney DM, Algaba F, Camparo P, Comperat E, Griffiths D, Kristiansen G, Lopez-Beltran A, Montironi R, Varma M, Egevad L (2013) The reasons behind variation in Gleason grading of prostatic biopsies: areas of agreement and misconception among 266 European pathologists. Histopathology 64(3): 405–411. 24102975 10.1111/his.12284

[bib2] Berney DM, Beltran L, Fisher G, North BV, Greenberg D, Møller H, Soosay G, Scardino P, Cuzick J on behalf of the transatlantic prostate group (2016) Validation of a contemporary prostate cancer grading system using prostate cancer death as outcome. Br J Cancer 114: 1078–1083. 27100731 10.1038/bjc.2016.86 PMC4865975

[bib3] Dong F, Yang P, Wang C, Wu S, Xiao Y, McDougal WS, Young RH, Wu CL (2013) Architectural heterogeneity and cribriform pattern predict adverse clinical outcome for Gleason grade 4 prostatic adenocarcinoma. Am J Surg Pathol 37(12): 1855–1861. 24145642 10.1097/PAS.0b013e3182a02169

[bib4] Epstein JI (2010) An update of the Gleason grading system. J Urol 183(2): 433–440. 20006878 10.1016/j.juro.2009.10.046

[bib5] Epstein JI, Egevad L, Amin MB, Delahunt B, Srigley JR, Humphrey PA, Grading C (2016) The 2014 International Society of Urological Pathology (ISUP) consensus conference on Gleason grading of prostatic carcinoma: definition of grading patterns and proposal for a new grading system. Am J Surg Pathol 40(2): 244–252. 26492179 10.1097/PAS.0000000000000530

[bib6] Kir G, Sarbay BC, Gumus E, Topal CS (2014) The association of the cribriform pattern with outcome for prostatic adenocarcinomas. Pathol Res Pract 210(10): 640–644. 25042388 10.1016/j.prp.2014.06.002

[bib7] Kweldam CF, Wildhagen MF, Steyerberg EW, Bangma CH, van der Kwast TH, van Leenders GJ (2014) Cribriform growth is highly predictive for postoperative metastasis and disease-specific death in Gleason score 7 prostate cancer. Mod Pathol 28(3): 457–464. 25189638 10.1038/modpathol.2014.116

[bib8] Loeb S, Folkvaljon Y, Robinson D, Lissbrant IF, Egevad L, Stattin P (2016) Evaluation of the 2015 Gleason grade groups in a nationwide population-based cohort. Eur Urol 69(6): 1135–1141. 26707871 10.1016/j.eururo.2015.11.036 PMC4909574

